# Genome Sequences of Four Cluster P Mycobacteriophages

**DOI:** 10.1128/genomeA.01101-17

**Published:** 2018-01-11

**Authors:** Erin L. Doyle, Christy L. Fillman, Nathan S. Reyna, Deborah M. Tobiason, Daniel E. Westholm, Jonathan L. Askins, Brittany P. Backus, Ashlynn C. Baker, Harrison S. Ballard, Paul J. Bisesi, Logan Bond, Deanna Byrnes, Hannah Carlstedt, Kinnon S. Dodson, Megan J. Fallert, Kyla J. Foster, Daniel N. Games, Tristan R. Grams, Nancy A. Guild, Autumn Hurd, Nicholas Iwata, Cassidy R. Kepler, Lucinda R. Krenzke, Kelly Luekens, Jackie Lewis, Cali McEntee, Justin C. McGee, Noah Nalley, Ruth C. Plymale, Jade Prochaska, Reid G. Rogers, Jessica B. Schipper, Kelsey Snyder, Kali Uhrich, Chelsey D. Vermillion, Sarah K. Vickers, Meredyth D. Wenta, Tyler Z. Yates, Chas F. Young, Ty H. Stoner, Welkin H. Pope, Deborah Jacobs-Sera, Rebecca A. Garlena, Daniel A. Russell, Steven G. Cresawn, Graham F. Hatfull

**Affiliations:** a Biology Department, Doane University, Crete, Nebraska, USA; b Department of Molecular, Cellular and Developmental Biology, University of Colorado, Boulder, Colorado, USA; c Biology Department, Ouachita Baptist University, Arkadelphia, Arkansas, USA; d Biology Department, Carthage College, Kenosha, Wisconsin, USA; e Biology Department, The College of St. Scholastica, Duluth, Minnesota, USA; f Department of Biological Sciences, University of Pittsburgh, Pittsburgh, Pennsylvania, USA; g Department of Biology, James Madison University, Harrisonburg, Virginia, USA

## Abstract

Four bacteriophages infecting *Mycobacterium smegmatis* mc^2^155 (three belonging to subcluster P1 and one belonging to subcluster P2) were isolated from soil and sequenced. All four phages are similar in the left arm of their genomes, but the P2 phage differs in the right arm. All four genomes contain features of temperate phages.

## GENOME ANNOUNCEMENT

A large collection of over 1,400 sequenced bacteriophages infecting *Mycobacterium smegmatis* mc^2^155 reveals that they have considerable genetic diversity (1). Phages grouped in clusters N and P are temperate but use an unusual integration-dependent immunity system, in which *attP* is positioned within the repressor gene (2); genome integration results in the truncation of the repressor, removal of a C-terminal degradation tag, and expression of stable repressor protein.

Mycobacteriophages Bartholomew, Bogie, Ksquared, and Tortellini were isolated from soil samples from Kenosha, Wisconsin, USA; Camden, Arkansas, USA; Crete, Nebraska, USA; and Boulder, Colorado, USA, respectively, via enrichment cultures and plaque purification. Bartholomew and Bogie produced plaques with large halos and clear centers, while Ksquared and Tortellini produced turbid plaques. All four phages have siphoviral morphologies.

Purified DNA was sequenced using the Illumina MiSeq platform, and 140-bp single-end reads were assembled into a major contig for each phage using Newbler, with at least 150-fold coverage. Genome lengths range from 46,484 bp to 49,658 bp (Table 1), and G+C% contents range from 65.8% to 67.2%, similar to the host bacterium (67.4%). All four genomes have defined ends with 12 base 3′ single-strand DNA extensions (Table 1). Bartholomew, Bogie, and Ksquared are closely related to each other and to other subcluster P1 phages at the nucleotide sequence level. Tortellini is closely related (>80% nucleotide identity) to these phages at the leftmost 40% of the genome but diverges in the rightmost 60% of the genome (<22% nucleotide identity). Tortellini is thus assigned as the first member of subcluster P2. Interestingly, although the rightmost 26-kbp segment of Tortellini has diverged considerably from subcluster P1 phages, the P1 phages have extensive sequence similarity to cluster I phages in this region (3). Genomes were auto-annotated using Glimmer (4) and GeneMark (5) and refined by manual inspection; the number of protein-coding genes ranges from 76 to 81 (Table 1). Functions were assigned using Phamerator (6), BLASTp (7), and HHpred (8). No tRNAs are present in any of the genomes.

**TABLE 1:** Genomic information for cluster P bacteriophages

Phage name	Accession no.	Genome size (bp)	GC%	No. of genes	ssDNA^a^ termini
Bartholomew	MF140399	46,484	67.2	77	CCCGCCCCCCGA
Bogie	MF133446	48,639	66.9	81	CCCGCCCCCCGA
Ksquared	MF281061	48,699	67.1	80	CCTGCCGCCCGA
Tortellini	KX648391	49,658	65.8	76	CCTGCCGCCCGC

a ssDNA, single-stranded DNA.

All four phages have features of temperate phages, including a tyrosine integrase, an immunity repressor, and an antirepressor. The three P1 phages contain integration-dependent immunity systems based upon the location of the *attP* site within their immunity repressor genes, overlapping a host tRNA-Thr^(CGT)^ gene, and SsrA-like degradation tags at the ends of the integrases and repressors (2). Similar systems are common in cluster P phages (3, 9). Tortellini is unusual among cluster P phages in that it does not have the characteristics of the integration-dependent immunity systems, and we could not identify a plausible *attP* site. A putative programmed frameshift is predicted to be involved in the expression of the tail assembly chaperone genes located upstream of the tape measure gene in all four phages. The three P1 phages have lysis cassettes with putative lysin A, lysin B, and holin genes, but Tortellini lacks a lysin B gene. In addition, the P1 phages code for a RecE/RecT system, an FtsK DNA translocase, and a RusA-like Holliday junction resolvase, which are not encoded in Tortellini.

### Accession number(s).

GenBank accession numbers are shown in Table 1.
